# A Porous Reservoir-Backed Boronate Gel Microneedle for Efficient Skin Penetration and Sustained Glucose-Responsive Insulin Delivery

**DOI:** 10.3390/gels8020074

**Published:** 2022-01-24

**Authors:** Siyuan Chen, Takuya Miyazaki, Michiko Itoh, Hiroko Matsumoto, Yuki Moro-oka, Miyako Tanaka, Yuji Miyahara, Takayoshi Suganami, Akira Matsumoto

**Affiliations:** 1 Research Institute for Biomaterials, Tech Institute for Advanced Materials, College of Materials Science and Engineering, Suqian Advanced Materials Industry Technology Innovation Center, NJTech-BARTY Joint Research Center for Innovative Medical Technology, Nanjing Tech University, Nanjing 212000, China; siyuan.chen@njtech.edu.cn; 2 Institute of Biomaterials and Bioengineering, Tokyo Medical and Dental University, Tokyo 101-0062, Japan; miyazaki.bsr@tmd.ac.jp (T.M.); mito.mem@tmd.ac.jp (M.I.); hiroko.matsumoto.bsr@tmd.ac.jp (H.M.); morooka.bsr@tmd.ac.jp (Y.M.-o.); miyahara.bsr@tmd.ac.jp (Y.M.); 3 Kanagawa Institute of Industrial Science and Technology, Ebina 213-0012, Japan; 4 Department of Molecular Medicine and Metabolism, Research Institute of Environmental Medicine, Nagoya University, Nagoya 464-8601, Japan; tanaka@riem.nagoya-u.ac.jp (M.T.); suganami@riem.nagoya-u.ac.jp (T.S.)

**Keywords:** phenylboronic acid, gel, glucose-responsive, microneedle, insulin, drug reservoir

## Abstract

Recently, phenylboronic acid (PBA) gel containing microneedle (MN) technology with acute and sustained glucose-sensitive functionality has attracted significant research attention. Herein, we report a polyvinyl alcohol(PVA)-coated MNs patch with an interconnected porous gel drug reservoir for enhanced skin penetration efficiency and mechanical strength. The hybrid MNs patch fabricated with a novel, efficient method displayed a “cake-like” two-layer structure, with the tip part being composed of boronate-containing smart gel attached to a porous gel layer as a drug reservoir. The porous structure provides the necessary structural support for skin insertion and space for insulin loading. The mechanical strength of the hybrid MNs patch was further enhanced by surface coating with crystallized PVA. Compared with MNs patches attached to hollow drug reservoirs, this hybrid MNs patch with a porous gel reservoir was shown to be able to penetrate the skin more effectively, and is promising for on-demand, long-acting transdermal insulin delivery with increased patient compliance.

## 1. Introduction

Insulin therapy remains one of the most important aspects of diabetes treatment, especially Type 1 and advanced Type 2 diabetes [1,2]. Usually, insulin therapy requires subcutaneous injections several times per day, which results in pain, infections, and decreased quality of life (QoL) [3,4]. Thus, there is a need for novel delivery methods for insulin that are noninvasive and convenient. In this regard, transdermal insulin delivery has attracted attention due to its advantages such as better patient compliance and avoiding the first-pass effect [5]. However, the molecular weight of human insulin (5808 D_a_) exceeds that of the threshold (less than 500 D_a_) for permeating the stratum corneum barrier [6]. To overcome this, various chemical or physical enhancement methods have been investigated, such as chemical or biochemical enhancers, electroporation, magnetophoresis, iontophoresis, etc. [7,8,9]. Among these, microneedle (MN) technology has attracted great interest due to its convenience, high delivery efficiency, low cost and painless administration [10]. MN patches contain needles that are sharper than hypodermic needles, with lengths of approximately several hundred micrometers, i.e., long enough to penetrate the stratum corneum without reaching blood vessels or nerves [11]. Therefore, MN patches can form microscale channels for efficient transdermal insulin delivery in a noninvasive way [12].

Another concern associated with insulin therapy is inconsistent glycemia control. Overdoses of insulin can result in hypoglycemia that may lead to behavioral and cognitive disturbance, coma, brain damage, and even death [3]. Smart insulin MN patches, encapsulating glucose-sensitive elements such as glucose oxidase (GO_x_) [13], glucose-binding proteins such as concanavalin A (Con A), and [14] phenylboronic acid (PBA) [15], negate this risk by mimicking the pancreas, secreting insulin in response to hyperglycemia [16]. For example, the reported GO_x_ polymeric nanoparticles embedded smart insulin MN patches are capable of providing on-demand insulin release; this is essential to achieve continuous glycemic control in a manner which is minimally dependent on patient compliance [17,18,19]. However, safety concerns associated with GO_x_, such as intolerance of long-term use, protein-denaturing issues during storage, the creation of toxic byproducts via reactions with glucose, and “burst-like” release behavior in response to hyperglycemia conditions, may affect its wide application [13,20,21,22]. Compared with protein-based glucose-sensitive elements, PBA has unique advantages. As a synthetic compound, it avoids problems of denaturing, and its interaction with glucose does not lead to the creation of any harmful byproducts [23]. In addition, the reversible glucose-binding characteristic guarantees the long-term functionality of PBA-based MNs, and the glucose sensitivity can be controlled by modifying the stereochemistry and electronic properties [24].

These features underline the potential of PBA in glucose sensing and self-regulated insulin delivery applications. Our group has designed a derivative of PBA bearing optimally electron-withdrawing para-carbamoyl and meta-fluoro substituents (4-(2- acrylamidoethylcarbamoyl)-3-fluorophenylboronic acid, A_m_ECFPBA), achieving a p*K*a value of about 7.2, i.e., close to physiological pH [25,26]. Since A_m_ECFPBA undergoes a glucose-dependent change in anionic boronate fraction, combining it with acrylamide hydrogel generates a surface localized, microscopically dehydrated “skin layer” in response to glucose. This “skin layer” is able to control the diffusion of the loaded insulin, and disappears upon hydration with an increase of glucose concentration. Thus, a “skin layer” driven insulin release control capacity allows the synthetic PBA gel to function as a self-regulated insulin delivery system [27]. We have further developed several types of boronate-containing hydrogel MNs exhibiting acute and long-term glucose-responsive insulin release control, together with improved safety and feasibility for large-scale production due to their enzyme- and nanoparticle-free characteristics [28,29,30].

Aspects which require further research are as follows: (i) the hollow structure of the drug reservoir attached to the boronate-containing smart hydrogel MNs considerably impacts their skin insertion efficiency; and (ii) limited drug loading capacity remains an issue common to all MNs [31]. The strategy of attaching a drug reservoir to the back of MNs is advantageous in terms of providing adequate drug loading for long-term use [31]; a hollow drug reservoir allows sufficient space for drug loading. However, its hollow nature may decrease the skin insertion efficiency, since the insertion force cannot be transferred to the majority of the needles located in the middle part.

Hence, here, we report a hybrid glucose-responsive insulin MNs patch attached to a porous gel drug reservoir for enhanced skin penetration efficiency. Three fabrication methods were investigated, and ultimately, a two-step photopolymerization method was selected to fabricate the MNs patch. The PBA-containing hydrogel constitutes the needle tips only, while the porous gel layer attached to the back serves as the drug reservoir. The interconnected porous structure of the porous gel reservoir provide space for insulin loading and ensure physical support, especially in the middle part of the MNs, where force is applied during use. Furthermore, the loaded liquid insulin solution could be transported to the needle tip by capillary action. To further improve the mechanical properties of the proposed device, the MNs patch was coated with a crystalized PVA layer by the dipping-drying method. This MNs patch, with a “cake-like”, two-layer structure, displayed acute and sustained self-regulated insulin delivery with significantly improved mechanical strength and skin insertion efficiency, which suggests its potential for diabetes management applications.

## 2. Results and Discussion

### 2.1. Gel Synthesis

**Figure 1:**
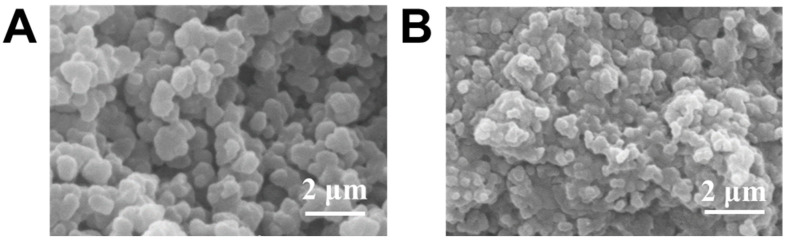
SEM image of (**A**) porous gel and (**B**) porous gel combined with glucose-responsive gel.

Porous gel was synthesized according to a method described in a previous publication [32]. A cross-section SEM image (Figure 1A) clearly shows the interconnected pores present throughout the gel after removing porogen PEG. This porous structure enabled rapid fluid transportation [33,34]. As a result, this porous matrix could be readily filled with the pregel solution (of glucose-responsive gel) due to its capillary action, followed by diffusion throughout the micropores [32]. After APS/TEMED-catalyzed radical copolymerization, the glucose-responsive gel formed within the porous structure, and the hybrid gel was obtained. Figure 1B shows the internal structure of the hybrid gel, which is remarkably different from that in Figure 1A. The SEM image proves the successful development of a hybrid, glucose-responsive porous gel, highlighting the benefits of the porous structure for both wetting and ease of diffusion.

### 2.2. Hybrid MNs Fabrication

The MNs patch was fabricated with widely-used micromolding technology [35]. The depth of the MN patch was designed to be 700 μm, i.e., enough to effectively and painlessly penetrate the stratum corneum but not so long as to touch nerve fibers and blood vessels.

**Figure 2:**
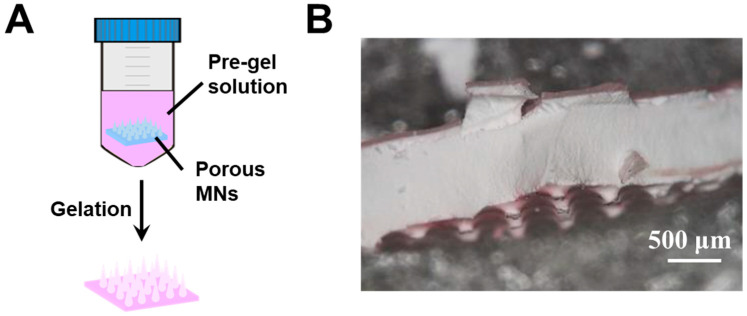
(**A**) Schematic of type 1 hybrid MNs fabricated by porous gel combined with glucose-responsive gel. (**B**) Cross-section morphology of type 1 hybrid MNs.

Type 1 hybrid MNs were fabricated by immersing the porous gel MNs in a glucose-responsive pregel solution (Figure 2A). The porous MNs displayed opaque color (Figure 2B) due to light scattering caused by the pores. Rhodamine B was added to the pregel solution to visualize the distribution of the glucose-responsive gel in the hybrid MNs. As seen in Figure 2B, although a pinkish color was observed in the internal structure, the majority of the glucose-responsive gel remained on the surface. This was due to the quick gelation which occurred during the APS/ TEMED-catalyzed radical copolymerization, i.e., the glucose-responsive gel formed on the surface before wetting and diffusing into the porous structure. Furthermore, the MNs tips formed by the porous gel were fragile, and broke when peeled off from the PDMS template. This affects the needle shape and further impacts skin penetration. Thus, another method was sought for hybrid MNs fabrication.

**Figure 3:**
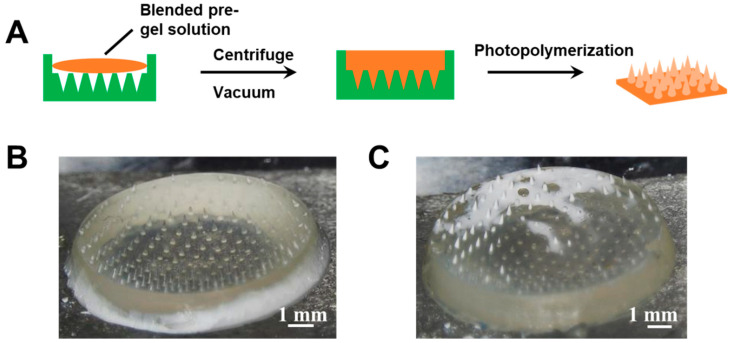
(**A**) Schematic of type 2 hybrid MNs fabricated by blended pregel solution. (**B**) Morphology of type 2 hybrid MNs fabricated by blending the pregel solution with a volume ratio of porous gel/glucose-responsive gel 5/5 (*v*/*v*). (**C**) Morphology of type 2 hybrid MNs fabricated by blending the pregel solution with a volume ratio of porous gel/glucose-responsive gel 6/4 (*v*/*v*).

The type 2 hybrid MNs patch was fabricated by one-step photopolymerization. The polymerization of glucose-responsive gel was initiated by photoinitiator Irgacure 184 instead of APS/TEMED. As shown in Figure 3, the pregel solutions of porous gel and glucose-responsive PBA gel were blended in various ratios and added to the PDMS template to allow MNs formation under UV light. Although conical needle shapes were observed in the MNs patch fabricated using this method, the distribution of the two gels was not homogenous (Figure 3B,C), which may affect the insulin release profile.

**Figure 4:**
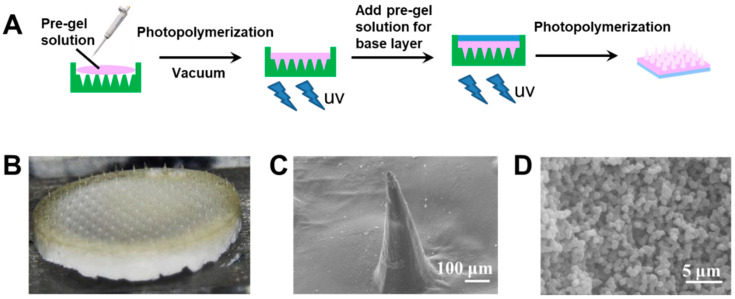
(**A**) Schematic of the type 3 hybrid MNs with a “cake-like” structure fabricated by two-step photopolymerization. (**B**) Morphology of the type 3 hybrid MNs with “cake-like” structure. (**C**) SEM image of the type 3 hybrid MNs tip part. Scale bar: 100 μm. (**D**) SEM image of the type 3 hybrid MNs base layer region. Scale bar: 5 μm.

The hybrid MNs fabrication method was further optimized. Two-step photopolymerization was applied to avoid inhomogeneous distribution of the two gels. Figure 4A shows a schematic of the fabrication by two-step photopolymerization of type 3 hybrid MNs. To ensure the quality of the needle molding and to control insulin release, the needle tip part was first formed by glucose-responsive gel (yellowish color in Figure 4B). After photopolymerization of the tip part, the pregel solution of porous gel was added to serve as the drug reservoir (i.e., the white base layer in Figure 4B). This “cake-like” structure was expected to achieve glucose-responsive insulin release, whilst the interconnected porous drug reservoir was intended to promote rapid fluid transportation with enhance mechanical strength. The conical needle shape (Figure 4C) and porous base layer structure (Figure 4D) were confirmed by SEM images.

### 2.3. Mechanical Strength and Skin Penetration

**Figure 5:**
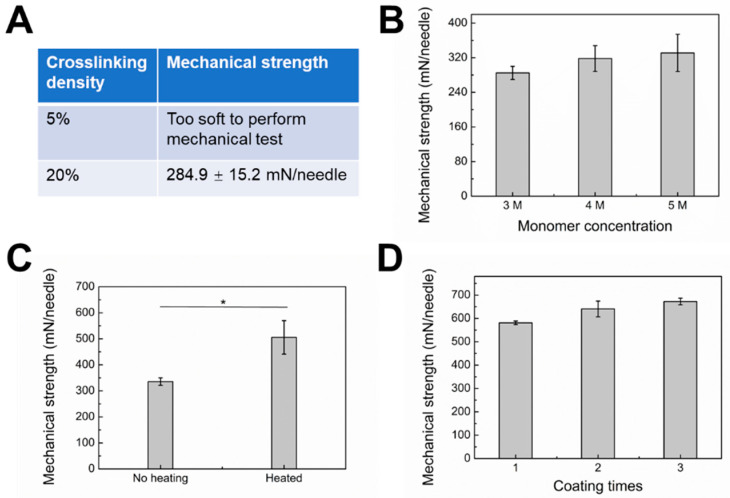
(**A**) Mechanical strength of “cake-like” microneedle tip part fabricated by glucose-responsive gel with different crosslinking densities. Monomer concentration: 3 M. (**B**) Mechanical strength of “cake-like” microneedle tip part fabricated by glucose-responsive gel with different monomer concentrations. Crosslinking density: 20%. (**C**) Mechanical strength of “cake-like” microneedle tip part coated with PVA layer with or without heating. * *P* < 0.05. (**D**) Mechanical strength of “cake-like” microneedle tip part coated with PVA layer with different coating times.

Relatively low mechanical strength limits the wide application of hydrogel MNs [36]. To increase the mechanical strength, one strategy is to fabricate MNs with a dense polymer network. As seen in Figure 5A,B, with increasing crosslinking density and monomer concentration, the mechanical strength increased accordingly. These results imply that a tight polymer network could result in MNs with increased mechanical strength. Meanwhile, the gel formulation must be carefully optimized to avoid decreased drug release rate with increased crosslinking density and monomer concentration. Another strategy is surface coating with materials with ideal mechanical strength. PVA is a widely used material for dissolving MNs due to its excellent biocompatibility and hydrophilicity [37]. Our MNs could be easily coated with PVA by the dipping-drying method. Notably, the aqueous stability and stiffness of PVA can be dramatically increased with heat treatment due to heat-induced crystallization [30]. After heat treatment, the mechanical strength of the type 3 hybrid MNs surface coated with PVA increased from 335.6 ± 14.2 to 505.6 ± 64.5 mN/needle (Figure 5C). The mechanical strength of PVA-coated type 3 hybrid MNs was further enhanced by increasing coating times. By repeating the coating and heating process three times, the MNs patch yielded 672.6 ± 14.1 mN/needle, i.e., a 10.2-fold margin of safety over the force required for insertion into the skin (60 mN per needle) without breakages. No obvious morphological changes were observed with different coating times (Figure S1).

**Figure 6:**
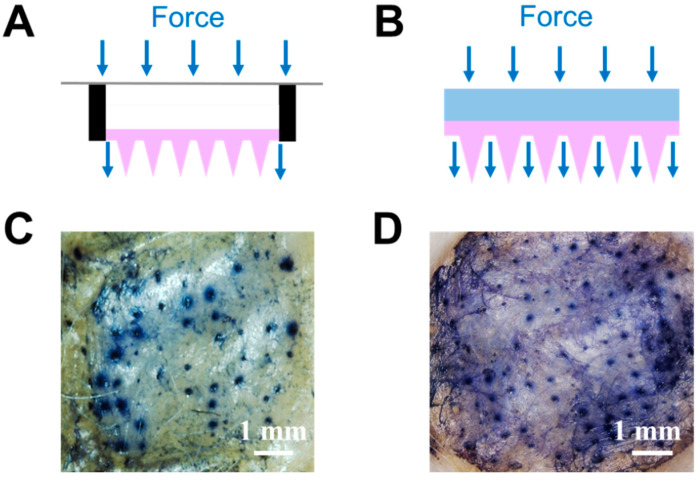
(**A**) Schematic of microneedles with a hollow drug reservoir, and (**B**) “cake-like” type 3 hybrid MNs with porous gel serving as a reservoir. Mouse skin was treated with (**C**) microneedles with a hollow drug reservoir or (**D**) “cake-like structure” type 3 hybrid MNs with porous gel reservoir for 10 min and stained with trypan blue to indicate the in vitro skin insertion ability.

Besides mechanical strength enhancement due to the optimized gel formulation and PVA coating, the porous drug reservoir design also increased skin insertion efficiency. Figure 6A shows a schematic of a previously reported drug reservoir attached to the back of MNs [28,30]. Due to its hollow design, the MNs patch functioned like a diaphragm; consequently, the force applied to the back of the patch was concentrated at the edge, with little force being transferred to the needles in the middle area. In contrast, the newly designed reservoir with an interconnected porous structure helped to achieve more homogeneous force distribution (Figure 6B). Thus, every needle should be subjected to a similar level of pressure, leading to enhanced skin insertion efficiency. Figure 6C,D compare the skin insertion efficiency between the two types of reservoirs. The skin penetration efficiencies were 65% (Figure 6D) and 30% (Figure 6C), respectively. The conical needles remained intact after skin insertion (Figure S2).

### 2.4. Glucose Responsive Insulin Release

**Figure 7:**
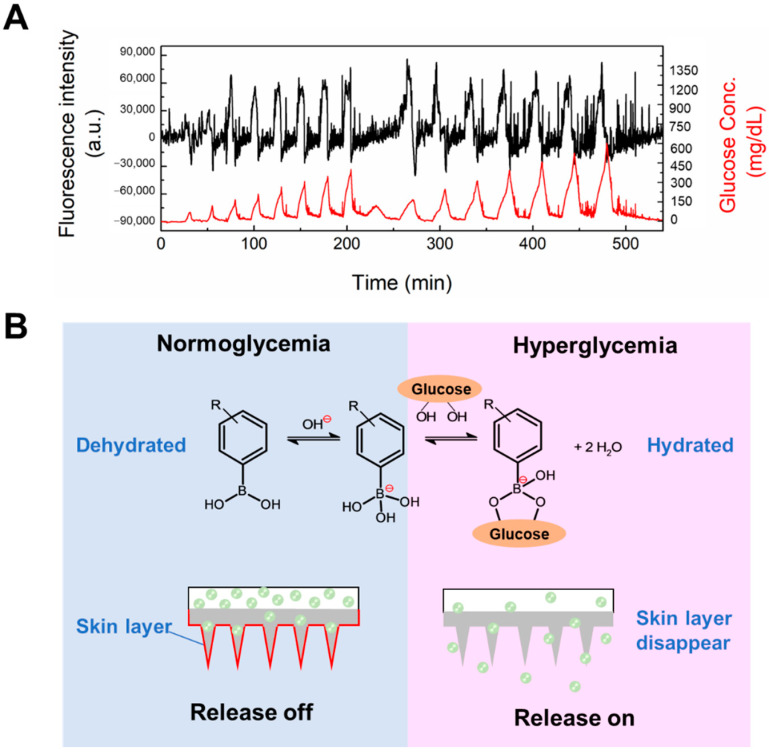
(**A**) In vitro glucose-responsive insulin release experiment at pH 7.4 and 37 °C. Time-course changes in the fluorescence intensity of FITC-labeled insulin released from microneedle with a “cake-like” structure (black line). Temporal patterns of the fluctuation in glucose concentration (red line). Initial equilibration was performed until no leakage of insulin was found. (**B**) Glucose-dependent equilibria of PBA derivatives and schematic representation of skin-layer-regulated glucose-responsive insulin release.

The increased monomer concentration and crosslinking density, different polymerization methods and surface coating with a PVA layer would obviously affect the functionality of the gel. Hence, it was important to verify the glucose-responsive insulin release capability of the type 3 hybrid MNs. Insulin was added to the porous drug reservoir of the PVA-coated MNs patch, and the back and side parts of the porous reservoir were sealed with water-proof epoxy resin (silicone one-component room temperature vulcanizing rubber) to avoid leakage. This insulin-loaded type 3 hybrid MNs patch was challenged with various glucose patterns (Figure 7A. As seen in Figure 7A, the release of insulin could be controlled according to the glucose pattern for at least 9 h. This result suggests that in type 3 hybrid MNs, a “skin-layer” still formed under normoglycemia and hypoglycemia conditions which regulated the glucose-responsive insulin release. Figure S3 shows the MNs patch still maintained its original needle structure after challenged with various glucose patterns and release loaded insulin for 9 h. 

Consistent with the glucose-dependent shift in the equilibria of PBA (Figure 7B), the surface of the PBA gel-containing MNs was found to change rapidly, from a swollen hydrophilic state to a shrunken hydrophobic state, upon abrupt decrease in glucose concentration, forming a thin surface layer with collapsed polymer network [25,27,38]. This dehydrated surface layer, or “skin layer”, was able to control the diffusion of loaded insulin molecules, and thus, shut down release under hypoglycemia conditions. With an increase of glucose concentration and binding with PBA, the hydration of the PBA gel led to the disappearance of the skin layer, thereby restoring insulin release. By fine-tuning the chemical structure of the PBA and gel, the glucose concentration at which the skin layer formed was brought to normal blood glucose levels, thus allowing effective insulin release control under physiologically relevant conditions [26]. This “skin-layer” controlled release mechanism offers unique advantages, such as remarkably shortened response time, reversibility, and continuous insulin diffusion control, compared to methods based on other glucose-sensitive elements.

## 3. Conclusions

Herein, we described a PVA-surface-coated, glucose-responsive insulin-delivering MNs patch attached to a porous gel back-layer serving as a drug reservoir for improved mechanical strength and skin penetration.

Among three fabrication methods, two-step photopolymerization was found to be the most promising. In this method, the tip region was formed by the PBA-containing smart hydrogel, and the base layer was synthesized by the porous gel with interconnected pores. This porous structure left enough space for insulin loading and rapid fluid transportation by capillary action and diffusion. Furthermore, compared to a previously designed drug reservoir with a hollow structure, the porous gel layer provided structural support when applying force to the back of the MNs patch. The mechanical strength of the patch was further improved by optimizing the monomer concentration and crosslinking density of the PBA-containing hydrogel, as well as the PVA surface coating method. The PVA-coated MNs with porous gel layer displayed significantly improved skin penetration efficiency compared to MNs attached to hollow drug reservoirs. More importantly, despite the PVA surface coating, neither the modified gelation protocol nor the attachment of the porous gel layer interfered with the glucose-responsive functionality of the MNs patch. Acute and long-lasting glucose-responsive insulin release was observed, suggesting the potential of this device to achieve persistent glycemic control for diabetic patients in a painless and convenient way.

## 4. Materials and Methods

### 4.1. Materials

Trimethylolpropane trimethacrylate (TRIM), triethyleneglycol dimethacrylate, polyethylene glycol (10 kD_a_), 2-methoxyethanol, glycidylmethacrylate, Irgacure 184, N-Isopropylacrylamide (NIPAA_m_), ammonium persulfate (APS), tetramethyl-ethylenediamine (TEMED), 4-(2-acrylamidoethylcarbamoyl)-3-fluorophenylboronic acid (A_m_ECFPBA) and methanol, *N*,*N*′-methylenebis- (acrylamide) (MBAA_m_) were purchased from Wako Pure Chemical Industries (Tokyo, Japan). Poly(vinyl alcohol) (M_w_ 13,000–23,000, 98% hydrolyzed) and fluorescein isothiocyanate (FITC)-labeled bovine insulin were purchased from Sigma-Aldrich (Tokyo, Japan). Mouse skin was purchased from Hoshino Laboratory Animals, Inc., Bandou-shi, Japan. Waterproof epoxy resin KE-3424-G was obtained from Shin-Etsu Chemical Co. Ltd., Tokyo, Japan.

### 4.2. Methods

#### 4.2.1. Synthesis of Porous Gel

**Scheme 1:**
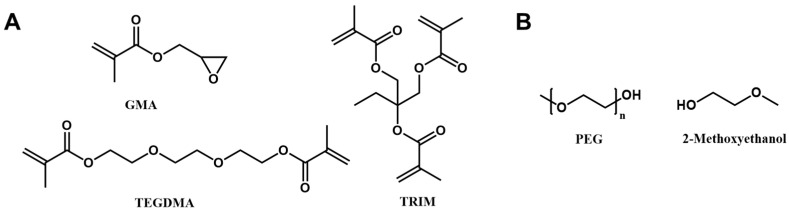
(**A**) Monomer and crosslinker, and (**B**) porogen of the porous gel.

Porous gel with PEG as a porogen was synthesized according to the method described in a previous publication (Scheme 1) [32]. Briefly, PEG was dissolved in 2-methoxyethanol with a final concentration of 20 wt%. The monomer solution was prepared by mixing glycidyl methacrylate, TRIM, and TEGDMA with a molar ratio of 1:0.26:0.79. Before photopolymerization, the porogen solution was mixed with a monomer solution with a porogen ratio of 56%. Irgacure 184 (1 wt% to the monomer) was added to the reaction solution as a photoinitiator. The reaction was carried out under 365 nm UV light for 15 min. A porous structure was obtained by immersing the gel in methanol/water (1:1 volume) several times to remove the porogen PEG.

#### 4.2.2. Synthesis of Hybrid Glucose-Responsive Porous Gel

**Scheme 2:**
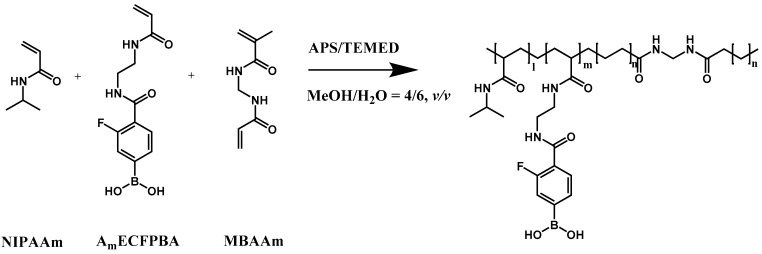
Chemical structure of glucose-responsive gel.

The glucose-responsive PBA gel reaction solution was prepared by dissolving NIPAA_m_ and A_m_ECFPBA in MeOH/H_2_O (4/6, *v*/*v*) with a fixed molar ratio of 92.5:7.5 and a total monomer concentration of 3 M. The cross-linker, MBAA_m_ was added to the pregel solution with 20 mol% monomer concentration. After the addition of 12 μL of 10 wt% APS and 12 μL of TEMED into 1 mL of reaction solution, the polymerization was initiated (Scheme 2). To synthesize the hybrid glucose-responsive porous gel, the porous gel with porogen removed was quickly immersed in the glucose-responsive PBA gel solution, vacuumed for 2 min to ensure the pregel solution entered the pores present throughout the porous gel, and then removed to allow polymerization to occur. Unreacted monomers were removed by washing in MeOH/H_2_O (4/6, *v*/*v*) several times. The internal structure of the hydrogels was visualized by scanning electron microscopy (SEM, JEOL, JSM-6000, Yamagata Prefecture, Japan).

#### 4.2.3. Type 1 Hybrid MNs Fabrication

Type 1 hybrid MNs were fabricated by micromolding technology with conical shape needle template (needle height 700 μm, pitch-to-pitch size 500 μm, patch diameter 1.2 cm). Pregel solution of the porous gel was added on the MNs template, vacuumed for 5 min to ensure that the reaction solution perfectly filled the MNs tip and base layer region, and photopolymerized under 365 nm UV light for 15 min. Once the porogen was removed and the MNs were totally dried, the MNs were immersed in the glucose-responsive PBA pregel solution containing TEMED/APS as an accelerator and initiator, and vacuumed for 2 min. Radical copolymerization of the glucose-responsive PBA gel was carried out at room temperature as described before. The unreacted monomers were removed by washing.

#### 4.2.4. Type 2 Hybrid MNs Fabrication

Type 2 hybrid MNs were fabricated by combining the porous gel and the glucose-responsive PBA gel. To ensure gelation under UV light, Irgacure 184 was added to the glucose-responsive PBA gel solution as an initiator, instead of APS and TEMED. The porous gel reaction solution was mixed with the glucose-responsive PBA reaction gel at a certain ratio, and photopolymerization was carried out under 365 nm UV light for 15 min. The unreacted monomers were removed by washing.

#### 4.2.5. Type 3 Hybrid MNs Fabrication

Type 3 hybrid MNs were fabricated in two steps. Firstly, the glucose-responsive PBA reaction gel with Irgacure 184 as a photoinitiator was added to the MNs template, vacuumed for 5 min, and photopolymerized under 365 nm UV light for 90 s. Then, the porous gel solution was added to the MNs template and photopolymerization continued for 15 min. The unreacted monomers were removed by washing.

#### 4.2.6. PVA Coating

The tip part of the type 3 hybrid MNs was coated with PVA by the tipping-drying method. The MNs needles were tipped with 10 wt% PVA solution, with residual solution being carefully removed by tissue paper. The samples were then left to dry overnight at room temperature. The following day, the PVA-coated MNs were placed in an oven at 130 °C for 1 h to induce crystallization of PVA. This coating and heating step was repeated several times, as needed.

#### 4.2.7. MNs Morphology Study

The morphology of the MNs was observed by optical microscope (Olympus, Japan) and SEM. The dried MNs sample was coated with a thin layer of gold (around 150 nm in thickness) to ensure sufficient conductivity. The gold-coated sample was titled at a certain angle as needed and visualized by SEM (JEOL, JSM-6000, Yamagata, Japan).

#### 4.2.8. Mechanical Strength

The mechanical strength of the MNs was evaluated by a bond tester (Dage 5000, Nordson, Manchester, UK). The MNs were fixed to the test platform by vacuum with a stainless steel testing probe placed 200 μm above the MNs base layer. During the test, the testing probe pressed horizontally against the needles with increasing force. The mechanical strength was recorded as the maximum loading force before needle fracture.

#### 4.2.9. Skin Penetration

The micropores resulting from MNs skin penetration were observed by staining MNs administrated (5 min) mouse skin with trypan blue for 30 min. The skin penetration efficiency was calculated by the following equation:

Skin penetration efficiency = Micropores created by MNs skin penetration/Total number of needles in one MNs patch × 100%

#### 4.2.10. In Vitro Release

The glucose-responsive release profile of the MNs was investigated by high-performance liquid chromatography (HPLC, JASCO HPLC system) with a Tricorn empty high-performance column (GE Healthcare, 50 mm length, inner diameter 10 mm). Insulin was loaded into the porous drug reservoir of the MNs patch coated with crystallized PVA by immersing the MNs in 130 mg/L FITC-insulin solutions and vacuuming for 5 min. The drug reservoir was sealed by a waterproof epoxy resin, i.e., KE-3424-G. The drug-loaded MNs patch was challenged with glucose at various concentrations (between 100 and 500 mg/dL) by passing a mixture of PBS and 100 mg/dL glucose solution through the two pumps of the HPLC system, controlled by the ChromNAV software (JASCO, Tochigi Prefecture, Japan). The flow rate was kept at 1 mL/min, and the MNs patch was equilibrated with PBS until no leakage was observed. The in vitro release of FITC-labeled insulin was monitored by determining the real-time fluorescence of FITC (E_x_ 490 nm, E_m_ 520 nm). The in situ glucose concentration was determined by the refractive index detector.

#### 4.2.11. Statistical Analysis

All measurements were taken in triplicate (*n* = 3). Reported results and graphical data are mean values with a standard deviation encompassing a 95% confidence interval.

## Data Availability

Data is contained within the article.

## Supplementary Materials

The following are available online at https://www.mdpi.com/article/10.3390/gels8020074/s1, Figure S1: The appearance of the type 3 MNs with “cake-like” structure (A) without PVA coating, (B) coated with PVA and heated once, (C) coated with PVA and heated twice and (D) coated with PVA and heated three times. Figure S2: The appearance of the type 3 MNs with a “cake-like” structure after skin insertion. Figure S3: The appearance of the type 3 MNs with a “cake-like” structure (A) before and (B) after HPLC experiment.
