# Supplementary material for: A Porous Reservoir-Backed Boronate Gel Microneedle for Efficient Skin Penetration and Sustained Glucose-Responsive Insulin Delivery
Source: Gels. 2022 Jan 24;8(2):74. doi: 10.3390/gels8020074 (PMC8871697; doi:10.3390/gels8020074)
Supplement: Supplementary file 1 [file gels-08-00074-s001.zip › gels-1522701-supplementary.pdf]

gels

Supplementary

A Porous Reservoir-Backed Boronate Gel Microneedle for Efficient Skin Penetration and Sustained Glucose-Responsive Insulin Delivery

Siyuan Chen [1], Takuya Miyazaki [2,3], Michiko Itoh [2,3], Hiroko Matsumoto [2,3], Yuki Moro-oka [2], Miyako Tanaka [4], Yuji Miyahara [2], Takayoshi Suganami [4] and Akira Matsumoto [2,3,*]

[1] Research Institute for Biomaterials, Tech Institute for Advanced Materials, College of Materials Science and Engineering, Suqian Advanced Materials Industry Technology Innovation Center, NJTech-BARTY Joint Research Center for Innovative Medical Technology, Nanjing Tech University, Nanjing,212000, China; siyuan.chen@njtech.edu.cn

[2] Institute of Biomaterials and Bioengineering, Tokyo Medical and Dental University, Tokyo, 101-0062, Japan; miyazaki.bsr@tmd.ac.jp (T.M.); mito.mem@tmd.ac.jp (M.I.); hiroko.matsumoto.bsr@tmd.ac.jp (H.M.); morooka.bsr@tmd.ac.jp (Yuki Moro-oka); miyahara.bsr@tmd.ac.jp (Yuji Miyahara)

[3] Kanagawa Institute of Industrial Science and Technology, Ebina, 213-0012, Japan

[4] Department of Molecular Medicine and Metabolism, Research Institute of Environmental Medicine, Nagoya University, Nagoya, 464-8601, Japan; tanaka@riem.nagoya-u.ac.jp (M.T.); suganami@riem.nagoya-u.ac.jp (T.S.)

* Correspondence: matsumoto.bsr@tmd.ac.jp

Citation: Chen, S.; Miyazaki, T.; Itoh, M.; Matsumoto, H.; Moro-oka, Y.; Tanaka, M.; Miyahara, Y.; Suganami, T.; Matsumoto, A. A Porous Reservoir-Backed Boronate Gel Microneedle for Efficient Skin Penetration and Sustained Glucose-Responsive Insulin Delivery. *Gels* **2022**, *8*, 74. https://doi.org/10.3390/gels8020074

Academic Editor: Damien Dupin

Received: 8 December 2021
Accepted: 10 January 2022
Published: 24 January 2022

Publisher's Note: MDPI stays neutral with regard to jurisdictional claims in published maps and institutional affiliations.

Copyright: © 2022 by the authors. Submitted for possible open access publication under the terms and conditions of the Creative Commons Attribution (CC BY) license (https://creativecommons.org/licenses/by/4.0/).

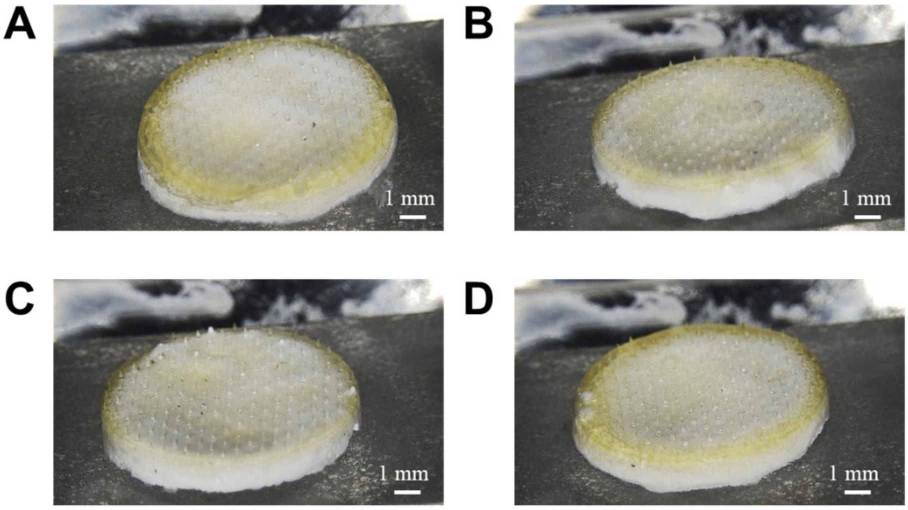

Figure S1. The appearance of the type 3 MNs with "cake-like" structure (**A**) without PVA coating, (**B**) coated with PVA and heated once, (**C**) coated with PVA and heated twice and (**D**) coated with PVA and heated three times.

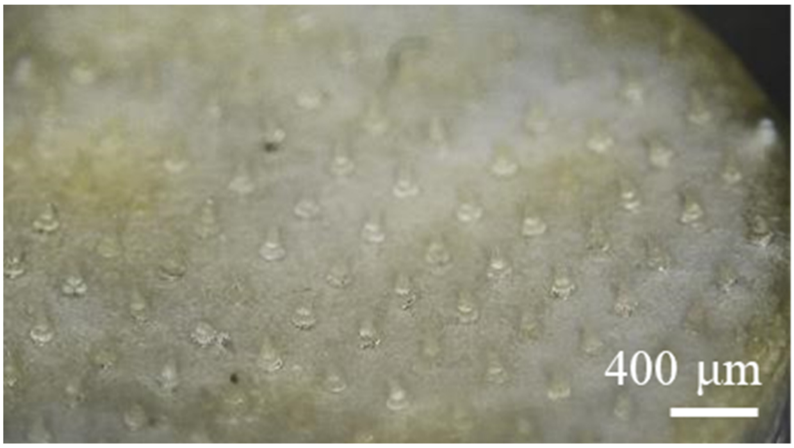

Figure S2. The appearance of the type 3 MNs with a "cake-like" structure after skin insertion.

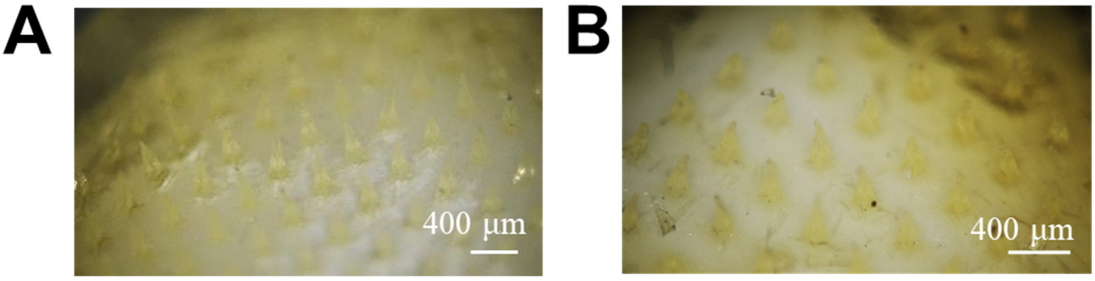

Figure S3. The appearance of the type 3 MNs with "cake-like" structure (**A**) before and (**B**) after HPLC experiment.
